# Neural mechanisms of infant learning: differences in frontal theta activity during object exploration modulate subsequent object recognition

**DOI:** 10.1098/rsbl.2015.0041

**Published:** 2015-05

**Authors:** Katarina Begus, Victoria Southgate, Teodora Gliga

**Affiliations:** Centre for Brain and Cognitive Development, Birkbeck College, University of London, Malet Street, London, WC1E 7HX, UK

**Keywords:** theta oscillations, learning, infants, motivation

## Abstract

Investigating learning mechanisms in infancy relies largely on behavioural measures like visual attention, which often fail to predict whether stimuli would be encoded successfully. This study explored EEG activity in the theta frequency band, previously shown to predict successful learning in adults, to directly study infants' cognitive engagement, beyond visual attention. We tested 11-month-old infants (*N* = 23) and demonstrated that differences in frontal theta-band oscillations, recorded during infants' object exploration, predicted differential subsequent recognition of these objects in a preferential-looking test. Given that theta activity is modulated by motivation to learn in adults, these findings set the ground for future investigation into the drivers of infant learning.

## 1. Introduction

Investigating predictors of learning success in infancy has relied largely on behavioural measures like visual attention. While termination of visual attention might indicate successful encoding [1], longer visual attention to stimuli does not necessarily predict better encoding or recognition at test [2]. This suggests that quality, rather than quantity, of attention may be more relevant for successful information processing. A promising means of elucidating how attentional quality supports learning in infancy is directly measuring the neural correlates, which have been shown to predict successful learning in adults.

A growing body of research is demonstrating that modulations in oscillatory activity in the theta frequency band (4–8 Hz in adults), believed to reflect prefrontal–hippocampal information-processing loops, correlate with memory performance at test. For example, Guderian *et al.* [3] demonstrated a linear relationship between power of theta activity before item presentation and rate of recall for those items at test. A similar relationship was found between prestimulus frontal theta activity and memory accuracy [4], as well as between frontal theta activity during retention and the capacity of visual working memory [5].

In infants, an increase in theta oscillations has been reported in situations often associated with infant learning, such as during periods of sustained attention [6], when infants were involved in a social game and exploration of novel objects [7], when infants' expectations were violated [8] and in response to infant-directed speech [9]. While some authors have interpreted theta oscillations as indexing implicit learning in infants [10], no study has so far directly explored whether theta oscillations in fact predict successful encoding in infants.

To address this, we recorded EEG activity while infants explored novel objects. Based on previous work demonstrating that object exploration induced the greatest modulation of theta oscillations over the frontal scalp location [7], we ranked the explored objects for each infant based on the power of frontal theta-band oscillations during exploration. We then tested infants' encoding of the objects' features in a preferential-looking task. We predicted that infants would learn more about the objects that were associated with more frontal theta-band activity; thus, differences in the power of frontal theta oscillations during exploration should be reflected in differences in infants' ability to discriminate the objects at test.

## 2. Material and methods

### (a) Participants

Twenty-three 11-month-old infants (13 female) were included in the sample; 12 infants were excluded owing to fussiness (4), insufficient data (4), parental interference (2) or experimental error (2).

### (b) Procedure

#### (i) Exploration phase

**Figure 1.**
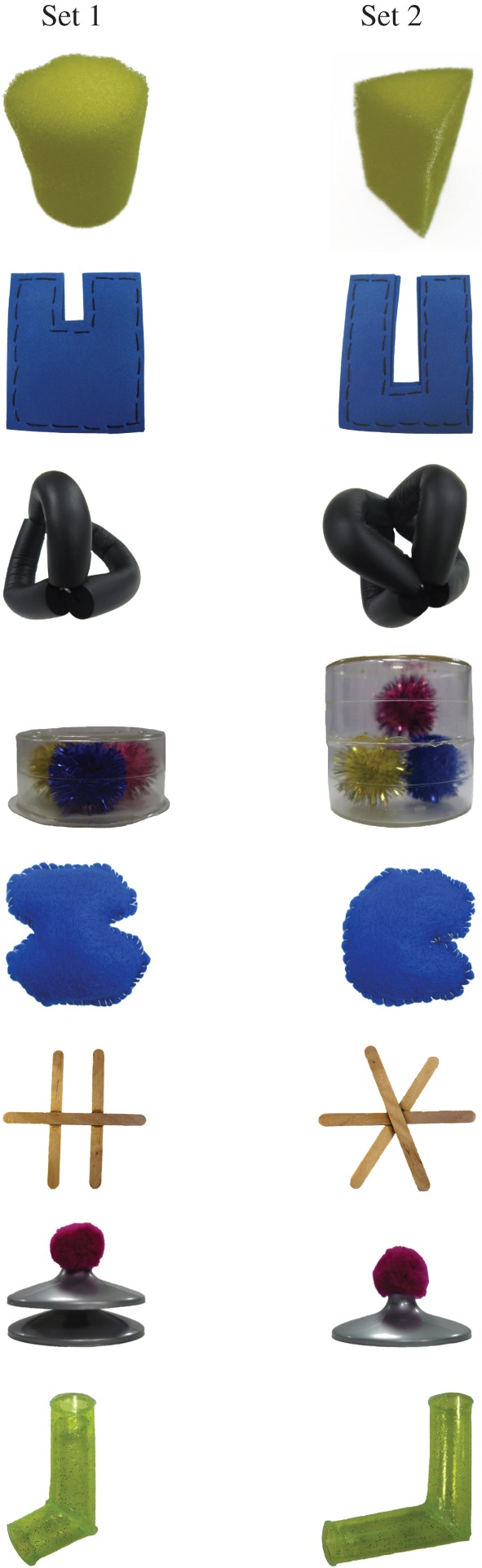
Novel objects. Infants explored all objects from one of the sets; images of pairs of objects from both sets were used as stimuli during Test phase. (Online version in colour.)

*Materials:* Infants were presented with one of two sets of eight novel objects (figure 1), approximately 10 × 10 cm in size and easily grasped and manipulated by infants. Each object in Set 1 was partially matched to one object in Set 2. Paired objects were matched in colour, size and material, but differed in shape (infants of this age can readily detect changes in shape [11]). Infants' behaviour was video recorded and their EEG was recorded at a sampling rate of 500 Hz using a 128-channel Geodesic Sensor Net (GSB; EGI Inc, Eugene, OR, USA).

*Procedure:* Infants sat in a high chair with a tray attached, on which each of the eight objects was presented individually, in a random order, for 40 s each. Each trial started with the sentence: ‘This is for you to play with’, and was preceded by a period (approx. 20 s) of blowing bubbles. The parent and the experimenter did not interact with the infant or objects, unless the object was dropped, in which case it was returned to the table immediately.

#### (ii) Test phase

*Materials:* Photographs of object pairs were displayed on a 102 × 58 cm plasma screen. When presented at 150 cm distance from the infant, each image subtended approximately the same visual angle as the physical object would during exploration. Infants' behaviour was video recorded.

*Procedure:* Infants were sat in a high chair or on their parent's lap. The parent was instructed not to interact with the infant. Trials started with an audio–visual animation in the centre of the screen to attract the infant's attention, followed by photographs of the familiar object (explored during Exploration phase) and the matched shape-distorted object (previously unseen), displayed side by side. Each trial lasted 12 s, with the side of presentation of the objects switching after 6 s.

### (c) Data analysis

#### (i) Exploration phase

**Figure 2.**
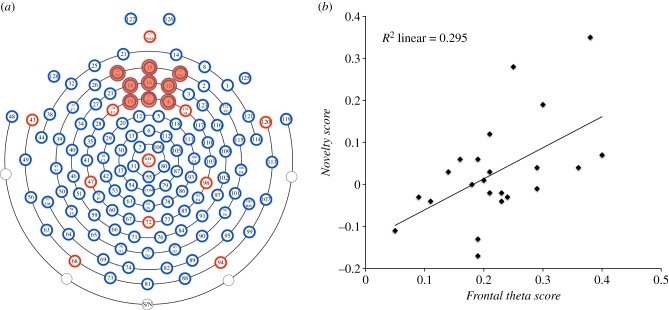
(*a*) EEG electrode map, with marked group of electrodes from which *Frontal theta score* data were extracted. (*b*) Relationship between *Frontal theta score* and *Novelty score*. (Online version in colour.)

*EEG analysis*: Video recordings were coded frame by frame (25 fps) and time intervals during which the infant was visually attending to the object were extracted for EEG analysis. The raw EEG data were imported to EEGlab, Fieldtrip, and visually screened for motion and eye-blink artefacts. Epochs of 1 s were then extracted from periods of continuous artefact-free data (any remaining samples were discarded; artefact-free segments were not concatenated but segmented separately) and fast Fourier transformed (Hanning window, 50% overlap) to yield a power spectrum between 1 and 50 Hz, in steps of 1 Hz. A minimum of 10 epochs of artefact-free data from a minimum of four objects was required for an infant's data to be analysed. Included objects were ranked according to the power of theta oscillations (3–5 Hz in infants [7]) measured at frontal central electrodes (figure 2*a*) during exploration. Two objects that elicited the highest (high theta objects (HTO)) and two objects that elicited the lowest power of theta oscillations (low theta objects (LTO)) were identified and averaged together. Difference scores were then calculated for each participant's EEG data (HTO − LTO/HTO + LTO), creating the variable *Frontal theta score.* Variable *Number of samples* was created to account for possible differences in the amount of data analysed for each object.

*Behavioural analysis*: To control for any variation in how infants interacted with the objects, which could lead to differences in encoding, video recordings of HTO and LTO explorations were coded for each infant's visual and manual exploration. Difference scores (HTO − LTO/HTO + LTO) were calculated to create variables *Visual exploration* (total looking time at the object, regardless of physical contact) and *Manual exploration* (total time the infant handled the object, while visually attending to it).

#### (ii) Test phase

Infants' looking behaviour was coded frame by frame (25 fps) to determine the magnitude of infants' looking-time preference for the presented objects (Novel − Familiar/Novel + Familiar). Trials in which the infant did not look for a minimum of 500 ms at each object presented on each side of the screen, were excluded from analysis. Looking-time difference scores were calculated for objects identified based on EEG data (HTO − LTO/HTO + LTO), creating the variable *Novelty score*. A score of 0 on this variable would mean the infant's looking-time preference was identical when discriminating HTO and LTO objects; a positive value would indicate that infants exhibited a larger novelty preference for HTO than LTO objects, and vice versa for a negative score.

## 3. Results

To establish whether a relationship exists between theta activity during exploration and infants' later recognition of the explored objects, a stepwise linear regression was performed on the data. To account for any variation in infants' exploration behaviour or amount of artefact-free data included in analyses, *Frontal theta score, Visual exploration*, *Manual exploration* and *Number of samples* were entered as predictors and *Novelty score* as the dependent variable. A significant model emerged (*F*_1,21_ = 8.803, *p* = 0.007, *R*^2^ = 0.295), explaining 29.5% of variance of the dependent variable. The only significant predictor of *Novelty score* was *Frontal theta score* (*β* = 0.543, *t*_21_ = 2.967, *p* = 0.007), whereas *Visual exploration*, *Manual exploration* and *Number of samples* did not explain a significant amount of variance and were therefore dropped from the model (multiple regression using *Enter* method produced the same results; see the electronic supplementary material for details). This relationship between *Frontal theta score* and *Novelty score* means that when the power of theta activity recorded during exploration was similar for HTO and LTO objects, these objects were similarly well (or poorly) discriminated at test (resulting in *Novelty score* values just below and above 0 (figure 2*b*)). Conversely, when the difference in theta activity between HTO and LTO objects was large, it was also reflected in a larger difference in infants' preferential looking, showing a stronger looking-time preference for HTO compared with LTO objects.

To examine whether our effect was specific to oscillations in the theta frequency band, the data were also analysed by ranking the objects based on power of oscillations in delta (1–3 Hz), alpha (6–8 Hz) and gamma (20–40 Hz) frequency bands over the frontal central electrodes. No significant relationship was found between *Novelty score* and power of oscillations in any other frequency band over the frontal central electrodes. In addition, further analysis revealed that the power of theta oscillations recorded over other scalp locations (occipital and temporal sites; electronic supplementary material, figure S1) did not significantly correlate with *Novelty score* (see the electronic supplementary material).

Note that while accounting for visual exploration, we could not control for potentially differential saccadic patterns during exploration. Altough this might be a caveat, previous findings showing within-trial modulations of saccadic amplitude in absence of modulation in the concurrently recorded frontal theta activity [12] suggest that an effect of saccades on our results is unlikely.

## 4. Discussion

This study is the first to demonstrate that modulations of frontal theta-band oscillations, recorded during infants' object exploration, predict infants' subsequent recognition of these objects. Specifically, the larger the difference between the power of theta activity recorded during exploration of two objects, the larger the difference in infants' subsequent recognition of these objects. The relationship found was specific to theta-band oscillations (3–5 Hz) recorded over the frontal cortex and was not present in any other frequency band or scalp area. Importantly, this relationship was not mediated by the length of infants' visual or manual exploration, suggesting that theta activity may provide a means of investigating infants' learning processes that cannot be captured by behavioural measures like visual attention.

While there is ample evidence that theta oscillations are involved in successful memory formation in adults, less is known about what drives the differences in the amount of theta activity for each individual. The timing and context dependency of theta activity in adult studies suggests that fluctuations in the power of theta are not random, but may reflect a strategic preparatory state for processing information [13]. Furthermore, it has been shown that theta activity can be modulated by expectancy of reward; only when participants were motivated to learn by monetary rewards did theta activity modulate recollection of words [4]. These findings are consistent with those of infant studies in which theta was recorded in situations where infants may expect to receive information, such as during infant-directed speech [9]; or be motivated to acquire new information, as in the case of violation of expectations [8].

Whether motivation modulates learning throughout life, including in infancy, remains largely unknown. Recent evidence that 16-month-olds use pointing to ask for information [14] and that information provided in response to pointing is better remembered [15] suggests the possibility that motivation drives learning even in infants. Theta activity, shown to be modulated by motivation in adults and demonstrated to be involved in learning in both adults and infants, could provide an important measure for investigating early behaviours suggested to signal interest or motivation to learn in infants, such as babbling and pointing [11,15], as well as what drives differential learning in the absence of behavioural differences. Finally, future research should also clarify whether differences in theta activity *between* individuals could explain individual differences in exploration and learning.

## Supplementary Material

Additional analyses

## Supplementary Material

Data
