# Supplementary material for: Neural mechanisms of infant learning: differences in frontal theta activity during object exploration modulate subsequent object recognition
Source: Biol Lett. 2015 May;11(5):20150041. doi: 10.1098/rsbl.2015.0041 (PMC4455734; doi:10.1098/rsbl.2015.0041)
Supplement: Additional analyses [file rsbl20150041supp1.docx]

**Table S1**. Additional analyses. Correlations between *Novelty score* and EEG activity in different frequency bands, recorded in the frontal electrode sites.

|  |  | ***Novelty score*** | |
| --- | --- | --- | --- |
|  | ***Frequency band*** | ***Pearson r*** | ***Sig. (2-tailed)*** |
| **Frontal electrodes** | Delta (1-3Hz) | 0.286 | 0.186 |
|  | Alpha (6-8Hz) | 0.223 | 0.307 |
|  | Gamma (20-40Hz) | 0.094 | 0.671 |

**Table S2.** Additional analyses. Correlations between *Novelty score* and Theta power recorded in other scalp locations.

| **Variable** | ***Beta in*** | ***t*** | ***Sig.*** |
| --- | --- | --- | --- |
| *Visual Exploration* | -0.196 | -0.985 | 0.336 |
| *Manual Exploration* | -0.178 | -0.858 | 0.401 |
| *Number of Samples* | -0.029 | -0.140 | 0.890 |

**Table S3.** Variables entered as predictors of *Novelty score* in the Stepwise linear regression (reported in manuscript), but were not included in the model.

| **Variable** | ***B*** | ***SE*** | ***Beta*** | ***t*** | ***Sig.*** |
| --- | --- | --- | --- | --- | --- |
| *(Constant)* | -0.157 | 0.070 |  | -2.234 | 0.038 |
| *Frontal Theta Power* | 0.805 | 0.293 | 0.585 | 2.746 | 0.013 |
| *Visual Exploration* | -0.350 | 0.374 | -0.401 | -0.936 | 0.362 |
| *Manual Exploration* | -0.093 | 0.269 | -0.141 | -0.345 | 0.743 |
| *Number of Samples* | 0.258 | 0.230 | 0.407 | 1.125 | 0.275 |

**Table S4.** Results of multiple regression analysis using the method ‘Enter’ (*F*=2.668, *p*=0.066, *R^2^*=0.372).

|  |  | ***Novelty score*** | |
| --- | --- | --- | --- |
|  | ***Scalp location*** | ***Pearson r*** | ***Sig. (2-tailed)*** |
| **Theta power** | Temporal Left | 0.269 | 0.215 |
|  | Temporal Right | 0.097 | 0.660 |
|  | Occipital | 0.085 | 0.701 |

**Figure S1**. Electrodes groups for the different scalp locations (FR = frontal, TL = Temporal Left, TR = Temporal Right, OC = occipital).
